# Association of Side-Branch Treatment and Patient Factors in Left Anterior Descending Artery True Bifurcation Lesions: Analysis from the GRAND-DES Pooled Registry

**DOI:** 10.1155/2020/8858642

**Published:** 2020-12-27

**Authors:** Gyu Chul Oh, Kyung Woo Park, Jeehoon Kang, Jung-Kyu Han, Han-Mo Yang, Hyun-Jae Kang, Bon Kwon Koo, Hyo-Soo Kim

**Affiliations:** Department of Internal Medicine, Cardiovascular Center, Seoul National University Hospital, Seoul 03080, Republic of Korea

## Abstract

**Methods:**

Patients undergoing PCI to left anterior descending (LAD) bifurcation lesions with contemporary DES were analyzed from a nationwide registry. Baseline risk was assessed using the Age, Creatinine, and Ejection Fraction (ACEF) score. Target lesion failure (TLF), a composite of cardiac death, target vessel myocardial infarction, and target lesion revascularization, was assessed at 3 years.

**Results:**

Among 1,089 patients with LAD bifurcation lesions, 548 (50.3%) patients underwent SB treatment. The SB treatment group showed a nonsignificant, but numerically lower rate of 3-year TLF (6.6% vs. 9.2%, HR 0.75, 95%CI 0.44–1.28, *p* = 0.29). In patients with low pretreatment risk (ACEF<1.22), SB treatment was associated with a lower rate of 3-year TLF (HR 0.43, 95%CI 0.19–0.96, *p* = 0.04), while no significant difference was observed in patients with high risk (ACEF≥1.22). The difference in the low risk group was mostly driven by target lesion revascularization (HR 0.24, 95%CI 0.08–0.75, *p* = 0.01).

**Conclusions:**

SB treatment for LAD bifurcation lesions showed favorable long-term outcomes compared with main-branch-only intervention, especially in patients with low pretreatment risk.

## 1. Introduction

Coronary bifurcation lesions are common and account for 15–20% of all percutaneous coronary interventions (PCI) [1]. Yet, they still pose a significant challenge to many interventional cardiologists. Bifurcation lesions are more complex in anatomy and lead to longer procedure time, requires more contrast volume and resources, and is associated with increased complications and low success rates [2].

Strategies for bifurcation PCI include a 1-stent approach, which is stenting of the main vessel (MV) first with provisional side-branch (SB) stenting, or an elective 2-stent approach. In the era of 1^st^ generation drug-eluting stents (DES), reports from randomized control studies (RCT) and pooled analyses have mostly favored the 1-stent approach. However, there is no concrete evidence behind this recommendation, especially in nonleft main (LM) coronary bifurcations. Furthermore, we do not know whether certain subgroup of patients may actually benefit from SB treatment. The previous studies mostly overlooked the clinical factors that may be important in future outcome of the bifurcation lesion. Analysis of a Korean bifurcation pooled cohort has shown that patients with diabetes were at high risk of adverse events, especially after 2 stenting in bifurcation PCI. Data from the European P2BiTO registry also reported that diabetes was associated with increased MACE after bifurcation PCI, regardless of treatment strategy [3].

The age, creatinine, and ejection fraction (ACEF) risk score was first introduced as a simple tool for predicting mortality in elective cardiac operations [4]. The three components in calculating the ACEF score, age, serum creatinine, and ejection fraction, are critical variables that must always be considered when planning and performing PCI. Although initially validated for short-term outcomes, it has also been applied to patients with acute coronary syndrome [5–7] and has been reported to be well correlated with long-term outcomes in all-comer PCI patients [8–10]. The ACEF score reflects the pretreatment risk of the patient and can be easily calculated.

We sought to evaluate the long-term outcomes of left anterior descending (LAD) true bifurcation lesions all treated with second-generation DES and whether the treatment of the SB may be of benefit in certain clinical risk groups, using a nationwide contemporary DES registry.

## 2. Materials and Methods

### 2.1. Study Design and Patient Population

**Figure 1:**
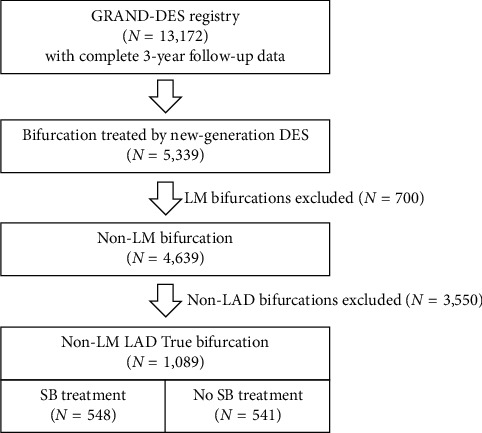
Study design. DES, drug-eluting stent; LM, left main coronary artery; LAD, left anterior descending coronary artery; ACEF, age, creatinine, and ejection fraction score; SB, side-branch; Tx, treatment.

The study population consists of patients from the Grand-DES registry, the details of which have been published in previous articles [11–13]. A total of 17,286 patients were enrolled at 55 participating centers from 2008 through 2014, and 13,172 patients treated with contemporary DES (5,154 with everolimus-eluting, 3,007 with biolimus-eluting, and 5,011 with zotarolimus-eluting stents) were screened for eligibility. Patients with LM disease and those without true bifurcation lesions were excluded, and 1,089 candidates who underwent PCI for left anterior descending (LAD) coronary true bifurcation lesions were selected for final analysis (Figure 1).

This study was conducted in accordance with the Declaration of Helsinki and has acquired the approval of the ethics committee of each participating center (ClinicalTrials.gov Identifier: NCT03507205). Written consent forms were acquired at the time of enrollment.

### 2.2. ACEF Score

The ACEF score was calculated using the following formula: ACEF = age/left ventricular EF + 1 (if creatinine >2.0 mg/dL) [4]. Patients with missing creatinine or left ventricular ejection fraction (LVEF) values were excluded in analyses using the ACEF score. Patients were divided into tertiles according to the calculated score, and outcomes were separately assessed in the lower two tertiles (ACEF-LOW; ACEF score <1.22) and highest tertile (ACEF-HIGH; ACEF score≥1.22).

### 2.3. Outcomes and Definitions

The primary outcome was target lesion failure (TLF), defined as a composite of cardiac death, target vessel myocardial infarction (TV-MI), and clinically driven target lesion revascularization (CD-TLR). Secondary outcomes were individual components of the primary outcome, all-cause death, all MI, any revascularization, target vessel revascularization, and stent thrombosis (ST). Individual outcomes were defined according to Academic Research Consortium definitions [14]. All patients had complete 3-year follow-up assessments, and outcomes were compared according to SB treatment and baseline risk obtained from ACEF scores.

True bifurcation lesions were defined as those having significant (>50%) stenosis in both the main branch and the SB (Medina 1, 1, 1; 1, 0, 1; or 0, 1, 1) [15]. Coronary intervention was performed according to current standard guidelines and techniques. Treatment strategy, including the number and type of stent, predilatation, adjunctive balloon inflation, and use of intravascular ultrasound (IVUS), was at the discretion of the operator. Side-branch treatment, unless otherwise specified, was defined as any predilatation, poststenting dilatation, kissing ballooning, or stenting of the SB. Treatment groups were further divided as either ballooning or stenting in subgroup analysis.

### 2.4. Statistical Analysis

Continuous variables are described as mean ± standard deviation (SD) and compared using Student's *t*-test or analysis of variance (ANOVA). Categorical variables are shown in numbers and percentages and compared using the chi-square test. Event rates for each outcome were calculated, and comparison of outcomes according to treatment strategy and baseline risk was performed using the log-rank test. Hazard ratios (HR) and 95% confidence intervals (CI) were calculated using Cox proportional hazard (PH) regression analysis. A multivariate Cox PH model was constructed to adjust for differences in baseline characteristics. The covariates used for multivariate Cox PH regression analysis are as follows: sex, 3-vessel disease, severe lesion calcification or tortuosity, and ACEF score. All *p* values were 2 sided, and *p* < 0.05 was considered to be significant. All analyses were performed using SPSS version 25 (IBM Crop., Armonk, NY, USA).

## 3. Results and Discussion

### 3.1. Baseline Characteristics

The baseline characteristics of the entire study population and those of the SB-treated and nontreated groups are described in Table 1. From a total of 1,089 patients, 548 (50.3%) underwent SB treatment. Patients in the SB-treated group were younger (63.9 ± 11.1 vs. 65.5 ± 10.6 years, *p* = 0.01) and had a lower prevalence of comorbidities and LV dysfunction. There were a higher proportion of patients with single LAD disease in the SB-treated group (40.5% vs. 26.2%, *p* < 0.01) and more calcified and tortuous lesions in the nontreated group. Among patients undergoing SB treatment, 373 (68.1%) underwent balloon angioplasty for the SB, while 175 (31.9%) performed SB stenting. Baseline characteristics for patients according to the type of SB treatment methods are shown in Supplementary Table S1.

The ACEF score was calculated for 904 patients with available creatinine and LVEF values at baseline. The score ranged from 0.49 to 5.36 (mean ± SD; 1.21 ± 0.48), with the SB nontreatment group showing a higher mean score compared to the treatment group (1.27 ± 0.54 vs. 1.15 ± 0.40, *p* < 0.01). As previously described, the ACEF score was used to group patients into low (ACEF-LOW) and high (ACEF-HIGH) pretreatment risk groups, with 1.22 as the cutoff value. The baseline characteristics of patients in the ACEF-LOW and ACEF-HIGH group are shown in Table 2 and Supplementary Table S2.

### 3.2. Clinical Outcomes

The complete results of the 3-year composite and individual clinical outcomes according to SB treatment and ACEF risk is presented in Table 3. In the whole study population, the rate of TLF was numerically lower in the SB treatment group (6.6 vs. 9.2%), although the difference was not statistically significant (log-rank *p* = 0.12) (Supplementary Figure S1). Significantly lower rates of cardiac death were observed in the SB treatment group in the unadjusted analysis (log-rank *p* = 0.02) (Supplementary Figure S2), but the difference was not statistically significant after adjustment for covariates (HR 0.59, 95% CI 0.27–1.28, *p* = 0.18).

**Figure 2:**
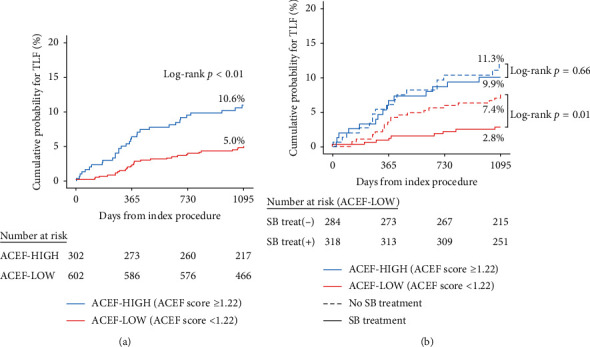
Kaplan–Meier curves for 3-year TLF according to the (a) ACEF risk group and (b) side-branch treatment. TLF, target lesion failure; ACEF, age, creatinine, and ejection fraction score; and SB, side branch.

**Figure 3:**
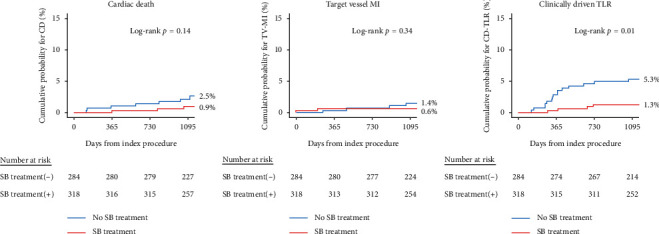
Kaplan–Meier curve for 3-year individual outcomes according to side-branch treatment in the ACEF-LOW group. MI, myocardial infarction; TLR, target lesion revascularization; and SB, side branch; ACEF, age, creatinine, and ejection fraction score.

The ACEF risk score was used to adjust for significant differences in baseline characteristics. The ACEF score correlated with observed outcomes, as ACEF-HIGH patients showed a significantly higher rate of TLF (Figure 2(a)). Although there was no difference in outcomes according to SB treatment in the ACEF-HIGH group, a significant reduction in 3-year TLF was observed with SB treatment in the ACEF-LOW group (Figure 2(b)). The rates of the individual outcomes were also numerically lower in the SB treatment group, with a significant lower rate of CD-TLR observed with SB treatment in the ACEF-LOW group (HR 0.24, 95% CI 0.08–0.75, *p* = 0.01). Individual outcomes for the ACEF-LOW group are presented in Figure 3. Clinical outcomes for the ACEF-HIGH group are presented in Supplementary Table S3.

Rates of stent thrombosis were low throughout the study population, with 0.5%/3 yr for the SB-treated group and 0.6%/3 yr for the nontreated group (Supplementary Figure S3). There was no significant difference according to SB treatment in both the low- and high-risk groups.

### 3.3. Type of SB Treatment

**Figure 4:**
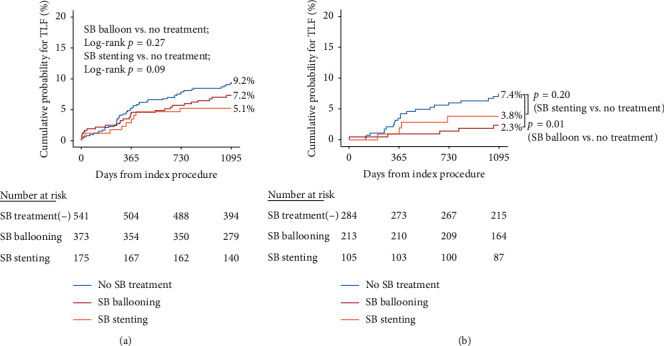
Kaplan–Meier curves for 3-year target lesion failure according to side-branch treatment method. (a) Whole study population. (b) ACEF-LOW group. SB, side branch. Predictors of composite outcome and post hoc analysis.

Side-branch treatment was further divided into either 2 stenting or SB ballooning. In the whole study population, the rate of TLF was numerically lower both in the 2-stent and SB-ballooning group compared with the nontreatment group, but without statistical significance (HR 0.77, 95% CI 0.42–1.41, *p* = 0.40 for SB ballooning vs. no treatment; HR 0.81, 95% CI 0.37–1.74, *p* = 0.58, SB stenting vs. no treatment). In the ACEF-LOW group, ballooning of the SB was associated with significantly lower rates of TLF compared with no treatment, but there was no significant difference between stenting and no treatment (Figure 4).

**Figure 5:**
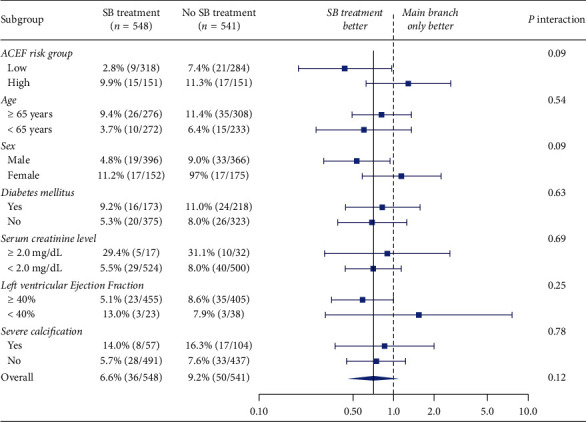
Subgroup analysis according to patient factors. SB, side branch; ACEF, age, creatinine, and ejection fraction score.

Independent predictors for TLF were assessed using multivariate Cox regression models. After adjusting for covariates, the predictors of poor outcome were increased ACEF score (HR 2.46, 95% CI 1.92–3.16, *p* < 0.01 for every 1-point increase) and severe lesion calcification (HR 1.43, 95% CI 1.51–3.90, *p* < 0.01) (Supplementary Table S4). In a post hoc subgroup analysis, there was no significant interaction for the primary outcome, except for ACEF risk and sex (Figure 5).

## 4. Discussion

In this analysis reporting the long-term outcomes of LAD bifurcation lesions using a prospective, nationwide registry, we observed that SB treatment was associated with a numerically lower rate of TLF compared with nontreatment. When stratified according to clinical risk, for those with low pretreatment risk according to the ACEF score, SB treatment significantly reduced 3-year TLF, mainly driven by the lower rate of revascularizations. Our analyses suggest that selected lower clinical risk patients may benefit from SB treatment and reduce the need for future revascularization and invasive procedures.

Numerous studies have reported that the 1-stent strategy, also known as the provisional SB stenting strategy, was associated with lower risk of adverse events. Thus, the current consensus for bifurcation PCI is to initially perform main vessel stenting, with provisional stenting of the SB [16]. Provisional stenting remains as an alternative during the procedure and is advised when flow is reduced and angiographic results are suboptimal. Elective 2 stenting is recommended in certain situations such as complex lesions with calcified SB, SB ostial lesions >5 mm from the carina, and in bifurcations with major SB [17, 18]. However, these recommendations mainly focus on angiographic characteristics and tend to overlook the clinical factors associated with the patient. We showed that the potential benefit of SB intervention may be slightly different according to the baseline clinical risk. The ACEF score, a composite of age, renal, and LV systolic function, was correlated with outcome and the benefit of SB intervention in bifurcation PCI.

Interestingly, TLF rates for patients in the high ACEF risk group were not affected by SB treatment, but there was a significant difference for the low-risk group. The results of individual outcomes show that this difference was mostly driven by CD-TLR, occurring mostly in the 6- to 18-month period, suggesting that proper full treatment of the SB at the time of the initial procedure might help in preventing future interventions.

The patients analyzed in the present study were exclusively those with newer generation DES. While the bulk of the data comparing one vs. two-stent strategy in bifurcation PCI was from the 1st generation DES era, newer generation DES have been reported to improve outcomes [19], and in studies comparing 2 stenting between 1st and 2nd generation DES, newer DES showed significantly superior outcomes to the older generation DES [20]. Development of newer DES with thinner struts and improved deliverability has allowed for lower restenosis and higher success rates, lessening the gap between SB treatment strategies. In our analysis, 3-year TLF was numerically lower in the 2-stent group. Furthermore, this trend was consistent regardless of the individual component of TLF. Similarly, in an all-comers randomized trial evaluating the effect of final kissing ballooning and 2 stenting using newer DES, 1-year angiographic and clinical outcomes were excellent in all groups once the procedure was successfully performed [21].

For patients with SB treatment, 16.1% underwent PCI with 2 stents, and 34.3% underwent balloon angioplasty of the SB with or without final kissing ballooning. No significant difference in outcomes was observed according to the type of SB treatment. For patients with low ACEF score, both balloon angioplasty and SB stenting were associated with a reduction in TLF compared with the non-SB treatment group. Balloon angioplasty of the SB, preferably followed by final kissing ballooning (FKB), could be an adequate option for patients with low pretreatment risk.

Outcomes of bifurcation PCI may rely on various patient, lesion characteristics, device selection, and operator-related factors. Intravascular ultrasound (IVUS) and optical coherence tomography (OCT) can visualize and calculate the complex anatomy of a bifurcation lesion, making it possible to predict SB compromise beforehand [22, 23]. Techniques such as proximal optimization (POT) and final kissing ballooning, jailed wire, and balloon methods have also allowed for better results in bifurcation PCI. Furthermore, physiologic assessment of the SB using fractional flow reserve (FFR) has also helped select which SB to treat [24].

The ACEF score is a simple, intuitive method that can be calculated in a matter of seconds. Simple and easily available clinical variables may help us guide our procedures. It has also been shown that the addition of clinical variables to the SYNTAX score, the so-called SYNTAX score II, improved the risk stratification capability of the score suggesting the importance of incorporating simple clinical variables into the decision-making process [25].

In the era of evidence-based medicine, adhering to treatment guidelines or expert opinions is important. However, for non-LM coronary bifurcation lesions, the evidence is scarce and sometimes contradictory. In line with current recommendations, most interventionists will probably leave the side-branch alone without definite flow compromise. The results of the current analysis suggest that patient factors such as age and renal function are powerful prognostic factors, and those with high pretreatment risk have unfavorable outcomes. However, complete revascularization might be a better option for those with low pretreatment risk, as they are more likely to receive additional revascularization procedures in the near future and lead to an extended period of antiplatelet therapy. Of course, one has to always keep in mind that routine practice of SB treatment can lead to longer procedure time, more wires, balloons, increased use of contrast, and radiation exposure. The interventionist should carefully anticipate the risk of SB compromise and know how to select the patient who will benefit from elective SB treatment.

## 5. Limitations

There are several limitations to the current study. First of all, the patient population was not derived from a bifurcation-dedicated registry and was a subgroup of a pooled DES registry. Criteria on whether to treat the side branch was not predefined, and the decision to treat a certain side branch relied heavily on the operator, which could have influenced outcomes. Second, there were significant differences in baseline characteristics between groups. Patients whose side branches were treated were generally younger, had less comorbidities, and were more likely not to have 3 vessel disease. Although a multivariate Cox Proportional Hazards model was used to adjust for differences, the results need to be interpreted with caution. Third, we were not able to assess whether SB treatment was a result of bail-out treatment (initial one-stent strategy but ended up being a two-stent procedure) or if it was a planned two-stent approach. Finally, there were no data on whether final POT was performed, a factor which is known to be associated with outcomes in bifurcation PCI.

## 6. Conclusions

For patients undergoing PCI of LAD bifurcation lesions, lower clinical risk patients may potentially benefit from complete treatment of the SB by reducing the need for future repeat revascularization.

## Figures and Tables

**Table 1:** Baseline characteristics according to SB treatment.

	Total (*n* = 1,089)	SB treatment (*n* = 548)	No SB treatment (*n* = 541)	*p* value
*Patient factors*				
Sex (male, %)	762 (70.0)	396 (72.3)	366 (67.7)	0.11
Age (years)	64.7 ± 10.9	63.9 ± 11.1	65.5 ± 10.6	0.01
BMI (kg/m^2^)	24.4 ± 3.1	24.4 ± 3.1	24.4 ± 3.1	0.88
Current smoker	301 (27.6)	157 (28.7)	144 (26.6)	0.50
*Comorbidities*				
Hypertension	669 (61.4)	318 (58.0)	351 (64.9)	0.02
Diabetes	391 (35.9)	173 (31.6)	218 (40.3)	<0.01
Dyslipidemia	668 (61.3)	333 (60.8)	335 (61.9)	0.74
CKD	48 (4.4)	17 (3.1)	31 (5.7)	0.04
CVA	91 (8.4)	46 (8.4)	45 (8.3)	1.00
PAD	15 (1.4)	8 (1.5)	7 (1.3)	1.00
FHx of CHD	86 (7.9)	47 (8.6)	39 (7.2)	0.47
Previous MI	48 (4.4)	24 (4.4)	24 (4.4)	1.00
Present with MI	306 (28.1)	162 (29.6)	144 (26.6)	0.31
ACS	647 (59.4)	320 (58.4)	327 (60.4)	0.53
LV dysfunction (EF<40%)	61 (6.6)	23 (4.8)	38 (8.6)	0.03
Serum Cr, mg/dL	0.9 (0.8–1.1)	0.9 (0.8–1.1)	0.9 (0.8–1.2)	0.07
History of PCI or CABG	109 (10.0)	52 (9.5)	57 (10.5)	0.64
*Lesion characteristics*				
3VD	333 (30.6)	139 (25.4)	194 (35.9)	<0.01
2VD	392 (36.0)	187 (34.1)	205 (37.9)	
1VD	364 (33.4)	222 (40.5)	142 (26.2)	
Medina class				0.96
1, 1, 1	798 (73.3)	401 (73.2)	397 (73.4)	
1, 0, 1	89 (8.2)	46 (8.4)	43 (8.0)	
0, 1, 1	202 (18.5)	101 (18.4)	101 (18.7)	
Calcified lesion	161 (14.8)	57 (10.4)	104 (19.2)	<0.01
Tortuous lesion	226 (20.8)	90 (16.4)	136 (25.1)	<0.01
*Procedure characteristics*				
SB balloon angioplasty	373 (34.3)	373 (68.1)	0 (0)	
SB stenting	175 (16.1)	175 (31.9)	0 (0)	
IVUS-guidance	471 (43.3)	250 (45.6)	221 (40.9)	0.03
GP IIb-IIIa inhibitor	48 (4.4)	23 (4.2)	25 (4.6)	0.13
*Medications*				
DAPT >1 year	575 (52.8)	281 (51.3)	294 (54.3)	0.34
RAS inhibitor	710 (65.2)	356 (65.0)	354 (65.4)	0.92
Beta-blocker	710 (65.2)	361 (65.9)	349 (64.5)	0.68
*ACEF score* ^*∗*^	1.21 ± 0.48	1.15 ± 0.40	1.27 ± 0.54	<0.01

Values are mean ± SD, median (interquartile ranges, 25^th^–75^th^), or *n* (%) (per-patient analysis). ^*∗*^Calculated in 904 patients with available values for creatinine and ejection fraction. SB, side-branch; BMI, body mass index; CVA, cerebrovascular accident; PAD, peripheral artery disease; FHx, family history; CHD, coronary heart disease; MI, myocardial infarction; ACS, acute coronary syndrome; LV, left ventricle; EF, ejection fraction; Cr, creatinine; PCI, percutaneous coronary intervention; CABG, coronary artery bypass graft surgery; 3VD, three-vessel disease; IVUS, intravascular ultrasound; GP, glycoprotein; DAPT, dual antiplatelet therapy; RAS, renin-angiotensin system; ACEF, age, creatinine, and ejection fraction.

**Table 2:** Baseline characteristics according to SB treatment in the low-risk group.

	ACEF-LOW (ACEF score <1.22)		*p* value
	SB treatment (*n* = 318)	No SB treatment (*n* = 284)	
*Patient factors*			
Sex (male, %)	236 (74.2)	199 (70.1)	0.30
Age (years)	59.7 ± 10.2	62.3 ± 9.6	<0.01
BMI (kg/m^2^)	24.6 ± 3.2	24.8 ± 3.0	0.47
Current smoker	100 (31.4)	86 (30.3)	0.83
Comorbidities			
Hypertension	182 (57.2)	174 (61.3)	0.36
Diabetes	95 (29.9)	104 (36.6)	0.10
Dyslipidemia	193 (60.7)	184 (64.8)	0.34
CVA	17 (5.3)	19 (6.7)	0.60
PAD	3 (0.9)	3 (1.1)	1.00
FHx of CHD	32 (10.1)	21 (7.4)	0.31
Previous MI	13 (4.1)	9 (3.2)	0.70
Presentation with MI	70 (22.0)	52 (18.3)	0.31
ACS	165 (51.9)	150 (52.8)	0.88
LV dysfunction (EF<40%)	2 (0.6)	1 (0.4)	1.00
Serum creatinine (mg/dL)	0.9 (0.8–1.1)	0.9 (0.8–1.1)	0.90
History of PCI or CABG	22 (6.9)	20 (7.0)	1.00
*Lesion characteristics*			
3VD	61 (19.2)	93 (32.7)	<0.01
Medina class			0.66
1, 1, 1	230 (72.3)	213 (75.0)	
1, 0, 1	25 (7.9)	23 (8.1)	
0, 1, 1	63 (19.8)	48 (16.9)	
Calcified lesion	30 (9.4)	47 (16.5)	0.01
Tortuous lesion	45 (14.2)	76 (26.8)	<0.01
*Procedure characteristics*			
SB balloon angioplasty	213 (67.0)	0 (0)	
SB stenting	105 (33.0)	0 (0)	
IVUS-guidance	159 (50.0)	126 (44.4)	0.19
GP IIb-IIIa inhibitor	11 (3.5)	6 (2.1)	0.45
*Medications*			
RAS inhibitor	204 (64.2)	179 (63.0)	0.84
Beta-blocker	209 (65.7)	178 (62.7)	0.49
*ACEF score*	0.94 ± 0.15	0.99 ± 0.14	<0.01

Values are mean ± SD, median (interquartile ranges, 25^th^-75^th^), or *n* (%) (per-patient analysis). SB, side branch; BMI, body mass index; CVA, cerebrovascular accident; PAD, peripheral artery disease; FHx, family history; CHD, coronary heart disease; MI, myocardial infarction; ACS, acute coronary syndrome; LV, left ventricle; EF, ejection fraction; PCI, percutaneous coronary intervention; CABG, coronary artery bypass graft surgery; 3VD, three-vessel disease; IVUS, intravascular ultrasound; GP, glycoprotein; RAS, renin-angiotensin system; ACEF, age, creatinine, and ejection fraction.

**Table 3:** Three-year composite and individual outcomes according to SB treatment.

Whole study population				ACEF-LOW group			
Outcomes (n, %)	SB treatment (*N* = 548)	No SB treatment (*N* = 541)	Adjusted HR†		SB treatment (*N* = 318)	No SB treatment (*N* = 284)	Adjusted HR†	
			HR (95% CI)	*p* value			HR (95% CI)	*p* value
Target lesion failure∗	36 (6.6)	50 (9.2)	0.75 (0.44–1.28)	0.29	9 (2.8)	21 (7.4)	0.43 (0.19–0.96)	0.04
Cardiac death	16 (2.9)	31 (5.7)	0.59 (0.27–1.28)	0.18	3 (0.9)	7 (2.5)	0.57 (0.14–2.32)	0.43
Target vessel MI	3 (0.5)	5 (0.9)	0.42 (0.07–2.42)	0.33	2 (0.6)	4 (1.4)	0.64 (0.11–3.75)	0.62
CD-TLR	17 (3.1)	20 (3.7)	0.75 (0.35–1.62)	0.46	4 (1.3)	15 (5.3)	0.24 (0.08–0.75)	0.01

Definite/probable ST	3 (0.5)	3 (0.6)	0.89 (0.07–11.83)	0.93	0	1 (0.4)	—	—
All-cause death	24 (4.4)	47 (8.7)	0.69 (0.38–1.27)	0.24	5 (1.6)	8 (2.8)	0.97 (0.30–3.08)	0.95
Any MI	6 (1.1)	7 (1.3)	0.78 (0.23–2.65)	0.70	4 (1.3)	5 (1.8)	0.89 (0.23–3.47)	0.87
Any repeat revascularization	56 (10.2)	50 (9.2)	1.12 (0.72–1.74)	0.62	25 (7.9)	29 (10.2)	0.82 (0.47–1.44)	0.49
Target vessel revascularization	28 (5.1)	30 (5.5)	0.96 (0.52–1.78)	0.90	10 (3.1)	20 (7.0)	0.48 (0.22–1.05)	0.07

^*∗*^Composite of cardiac death, target vessel MI, and CD-TLR. †Adjusted for sex, severe lesion calcification, tortuous lesion, 3-vessel disease, and ACEF score. SB, side branch; HR, hazard ratio; MI, myocardial infarction; CD-TLR, clinically driven target lesion revascularization; ST, stent thrombosis.

## Data Availability

The clinical and procedural data used to support the findings of this study may be released upon request and approval by the institutional review board of Seoul National University Hospital. Requests should be made to the corresponding author.

## Supplementary Materials

Supplementary Materials Supplementary Table S1. Baseline characteristics according to the side-branch treatment method. Supplementary Table S2. Patient characteristics according to ACEF risk. Supplementary Table S3. Three-year composite and individual outcomes according to SB treatment in the ACEF-HIGH group. Supplementary Table S4. Factors associated with TLF according to the multivariate Cox regression model. Supplementary Figure S1. Kaplan–Meier curve for 3-year TLF according to side-branch treatment. Supplementary Figure S2. Kaplan–Meier curve for 3-year individual outcomes according to side-branch treatment. Supplementary Figure S3. Kaplan–Meier curve for 3-year definite/probable stent thrombosis according to side-branch treatment.
