# Supplementary material for: Association of Side-Branch Treatment and Patient Factors in Left Anterior Descending Artery True Bifurcation Lesions: Analysis from the GRAND-DES Pooled Registry
Source: J Interv Cardiol. 2020 Dec 27;2020:8858642. doi: 10.1155/2020/8858642 (PMC7781708; doi:10.1155/2020/8858642)
Supplement: Supplementary Materials — Supplementary Table S1. Baseline characteristics according to the side-branch treatment method. Supplementary Table S2. Patient characteristics according to ACEF risk. Supplementary Table S3. Three-year composite and individual outcomes according to SB treatment in the ACEF-HIGH group. Supplementary Table S4. Factors associated with TLF according to the multivariate Cox regression model. Supplementary Figure S1. Kaplan–Meier curve for 3-year TLF according to side-branch treatment. Supplementary Figure S2. Kaplan–Meier curve for 3-year individual outcomes according to side-branch treatment. Supplementary Figure S3. Kaplan–Meier curve for 3-year definite/probable stent thrombosis according to side-branch treatment. [file 8858642.f1.docx]

**SUPPLEMENTARY MATERIAL**

# Association of side-branch treatment and patient factors in left anterior descending artery true bifurcation lesions: analysis from the GRAND-DES pooled registry

# Side-branch treatment in LAD bifurcations

Gyu Chul Oh,^1^ Kyung Woo Park,^1^ Jeehoon Kang,^1^ Jung-Kyu Han,^1^ Han-Mo Yang,^1^ Hyun-Jae Kang,^1^ Bon Kwon Koo,^1^ and Hyo-Soo Kim^1^

^1^ Department of Internal Medicine, Cardiovascular Center, Seoul National University Hospital, Seoul 03080, Republic of Korea.

Correspondence should be addressed to Kyung Woo Park; [kwparkmd@snu.ac.kr](mailto:kwparkmd@snu.ac.kr)

**Table of Contents Page**

Title page 1

Table of contents 2

Supplementary Tables 3-8

Supplementary Figures 9-11

**Table S1. Baseline characteristics according to side-branch treatment method.**

|  | SB  stent N=175 | SB  balloon N=373 | No SB treatment N=541 | P-value |
| --- | --- | --- | --- | --- |
| Patient factors |  |  |  |  |
| Sex (male, %) | 126 (72.0) | 270 (72.4) | 366 (67.7) | 0.25 |
| Age (years) | 63.9 ± 11.5 | 63.9 ± 11.0 | 65.5 ± 10.6 | 0.03 |
| BMI (kg/m^2^) | 24.3 ± 3.0 | 24.4 ± 3.2 | 24.4 ± 3.1 | 0.76 |
| Current smoker | 58 (33.1) | 99 (26.5) | 144 (26.6) | 0.21 |
| Comorbidities |  |  |  |  |
| Hypertension | 94 (53.7) | 224 (60.1) | 351 (64.9) | 0.03 |
| Diabetes | 61 (34.9) | 112 (30.0) | 218 (40.3) | 0.01 |
| Dyslipidemia | 107 (61.1) | 226 (60.6) | 335 (61.9) | 0.92 |
| CVA | 20 (11.4) | 26 (7.0) | 45 (8.3) | 0.21 |
| PAD | 5 (2.9) | 3 (0.8) | 7 (1.3) | 0.15 |
| FHx of CHD | 15 (8.6) | 32 (8.6) | 39 (7.2) | 0.71 |
| Previous MI | 7 (4.0) | 17 (4.6) | 24 (4.4) | 0.96 |
| Presentation with MI | 48 (27.4) | 114 (30.6) | 144 (26.6) | 0.42 |
| ACS | 11 (6.1) | 19 (4.3) | 327 (60.4) | 0.01 |
| LV dysfunction (EF<40%) | 10 (6.6) | 13 (4.0) | 38 (8.6) | 0.04 |
| Serum creatinine (mg/dL) | 0.9 [0.8, 1.1] | 0.9 [0.8, 1.1] | 0.9 [0.8, 1.2] | 0.10 |
| History of PCI or CABG | 11 (6.3) | 41 (11.0) | 57 (10.5) | 0.20 |
| Lesion characteristics |  |  |  |  |
| 3VD | 47 (26.9) | 92 (24.7) | 194 (35.9) | <0.01 |
| Medina class |  |  |  | 0.72 |
| 1,1,1 | 134 (76.6) | 267 (71.6) | 397 (73.4) |  |
| 1,0,1 | 11 (6.3) | 35 (9.4) | 43 (8.0) |  |
| 0,1,1 | 30 (17.1) | 71 (19.0) | 101 (18.7) |  |
| Calcified lesion | 23 (13.1) | 34 (9.1) | 104 (19.2) | <0.01 |
| Tortuous lesion | 30 (17.1) | 60 (16.1) | 136 (25.1) | <0.01 |
| Procedure characteristics |  |  |  |  |
| IVUS-guidance | 89 (50.9) | 161 (43.2) | 221 (40.9) | 0.07 |
| GP IIb-IIIa inhibitor | 7 (4.0) | 16 (4.3) | 25 (4.6) | 0.93 |
| Medications |  |  |  |  |
| RAS inhibitor | 111 (63.4) | 245 (65.7) | 354 (65.4) | 0.86 |
| Beta-blocker | 115 (65.7) | 246 (66.0) | 349 (64.5) | 0.89 |
| ACEF score | 1.15 ± 0.39 | 1.15 ± 0.40 | 1.27 ± 0.54 | <0.01 |

SB, side branch; BMI, body mass index; CVA, cerebrovascular accident; PAD, peripheral artery disease; FHx, family history; CHD, coronary heart disease; MI, myocardial infarction; ACS, acute coronary syndrome; LV, left ventricle; EF, ejection fraction; PCI, percutaneous coronary intervention; CABG, coronary artery bypass graft surgery; 3VD, three-vessel disease; IVUS, intravascular ultrasound; GP, glycoprotein; RAS, renin-angiotensin system; ACEF, age, creatinine, and ejection fraction.

**Table S2. Patient characteristics according to ACEF risk.**

|  | ACEF-LOW  (ACEF score<1.22)  N=602 | ACEF-HIGH  (ACEF score ≥1.22)  N=302 | P-value |
| --- | --- | --- | --- |
| Patient factors |  |  |  |
| Sex (male, %) | 435 (72.3) | 196 (64.9) | 0.03 |
| Age (years) | 60.9 ± 10.0 | 71.8 ± 9.2 | <0.01 |
| BMI (kg/m^2^) | 24.7 ± 3.1 | 23.7 ± 3.0 | <0.01 |
| Current smoker | 186 (30.9) | 58 (19.2) | <0.01 |
| Comorbidities |  |  |  |
| Hypertension | 356 (59.1) | 195 (64.6) | 0.13 |
| Diabetes | 199 (33.1) | 124 (41.1) | 0.02 |
| Dyslipidemia | 377 (62.6) | 177 (58.6) | 0.27 |
| CVA | 36 (6.0) | 35 (11.6) | 0.01 |
| PAD | 6 (1.0) | 6 (2.0) | 0.36 |
| FHx of CHD | 53 (8.8) | 20 (6.6) | 0.31 |
| Previous MI | 22 (3.7) | 16 (5.3) | 0.32 |
| Acute MI | 122 (20.3) | 126 (41.7) | <0.01 |
| ACS | 315 (52.3) | 204 (67.5) | <0.01 |
| LV dysfunction (EF<40%) | 3 (0.5) | 52 (17.2) | <0.01 |
| Serum creatinine (mg/dL) | 0.9 [0.8, 1.1] | 1.0 [0.8, 1.3] | <0.01 |
| History of PCI or CABG | 42 (7.0) | 35 (11.6) | 0.03 |
| Lesion characteristics |  |  |  |
| 3VD | 154 (25.6) | 115 (38.1) | <0.01 |
| Medina class |  |  | 0.28 |
| 1,1,1 | 443 (73.6) | 229 (75.8) |  |
| 1,0,1 | 48 (8.0) | 29 (9.6) |  |
| 0,1,1 | 111 (18.4) | 44 (14.6) |  |
| Calcified lesion | 77 (12.8) | 48 (15.9) | 0.24 |
| Tortuous lesion | 121 (20.1) | 63 (20.9) | 0.86 |
| Procedure characteristics |  |  |  |
| IVUS-guidance | 285 (47.3) | 123 (40.7) | 0.07 |
| GP IIb-IIIa inhibitor | 17 (2.8) | 14 ( 4.6) | 0.22 |
| Medications |  |  |  |
| RAS inhibitor | 383 (63.6) | 208 (68.9) | 0.14 |
| Beta-blocker | 387 (64.3) | 202 (66.9) | 0.48 |
| ACEF score | 0.97 ± 0.15 | 1.69 ± 0.53 | <0.01 |

ACEF, age creatinine ejection fraction; BMI, body mass index; CVA, cerebrovascular accident; PAD, peripheral artery disease; FHx, family history; CHD, coronary heart disease; MI, myocardial infarction; ACS, acute coronary syndrome; LV, left ventricle; EF, ejection fraction; PCI, percutaneous coronary intervention; CABG, coronary artery bypass graft surgery; 3VD, three-vessel disease; IVUS, intravascular ultrasound; GP, glycoprotein; RAS, renin-angiotensin system; ACEF, age, creatinine, and ejection fraction.

**Table S3. Factors associated with TLF according to multivariate Cox regression model.**

|  | *Univariate analysis* | | | *Multivariate analysis* | | |
| --- | --- | --- | --- | --- | --- | --- |
|  | **HR** | **95% CI** | **p-value** | **HR** | **95% CI** | **p-value** |
| Male | 0.64 | 0.41-0.98 | 0.04 |  |  |  |
| BMI (per 1kg/m^2^ increase) | 0.97 | 0.90-1.04 | 0.36 |  |  |  |
| Previous revascularization | 1.87 | 1.05-3.31 | 0.03 |  |  |  |
| Diabetes mellitus | 1.61 | 1.05-2.47 | 0.03 |  |  |  |
| Cerebrovascular accident | 1.93 | 1.05-3.55 | 0.04 |  |  |  |
| Hypertension | 2.24 | 1.35-3.74 | <0.01 |  |  |  |
| Peripheral artery disease | 3.31 | 1.05-10.48 | 0.04 |  |  |  |
| Severely calcified lesion | 2.43 | 1.51-3.90 | <0.01 | 2.97 | 1.73-5.11 | <0.01 |
| Presentation with ACS | 1.91 | 1.19-3.09 | 0.01 |  |  |  |
| 3 vessel disease | 1.72 | 1.12-2.65 | 0.01 |  |  |  |
| ACEF score | 2.46 | 1.92-3.16 | <0.01 | 2.49 | 1.92-3.23 | <0.01 |

HR, hazard ratio; CI, confidence interval; BMI, body mass index; ACS, acute coronary syndrome; ACEF, age, creatinine, and ejection fraction

**Table S4. 3-year composite and individual outcomes according to SB treatment in the ACEF-HIGH group.**

| Outcomes (n, %) | SB treatment  (N=151) | No SB treatment  (N=151) | Adjusted HR^†^ | |
| --- | --- | --- | --- | --- |
|  |  |  | **HR (95% CI)** | **p-value** |
| Target lesion failure* | 15 (9.9) | 17 (11.3) | 1.28 (0.62-2.67) | 0.51 |
| Cardiac death | 7 (4.6) | 16 (10.6) | 0.60 (0.24-1.51) | 0.28 |
| Target vessel MI | 0 (0.0) | 0 (0.0) | - | - |
| CD-TLR | 8 (5.3) | 1 (0.7) | 11.2 (1.35-92.82) | 0.03 |
| Definite/probable ST | 1 (0.7) | 1 (0.7) | 3.99 (0.12-128.84) | 0.44- |
| All-cause death | 12 (7.9) | 27 (17.9) | 0.59 (0.29-1.19) | 0.14 |
| Any MI | 1 (0.7) | 1 (0.7) | 0.68 (0.04-11.09) | 0.79 |
| Any repeat  revascularization | 20 (13.2) | 11 (7.3) | 1.93 (0.90-4.13) | 0.09 |
| Target vessel  revascularization | 11 (7.3) | 2 (1.3) | 6.73 (1.45-31.27) | 0.02 |

SB, side-branch; HR, hazard ratio; MI, myocardial infarction; CD-TLR, clinically driven target lesion revascularization; ST, stent thrombosis.

*****Composite of cardiac death, target vessel MI, and CD-TLR.

**†**Adjusted for sex, severe lesion calcification, tortuous lesion, 3-vessel disease, and ACEF score.

**Figure S1. Kaplan-Meier curve for 3-year TLF according to side-branch treatment.**

**
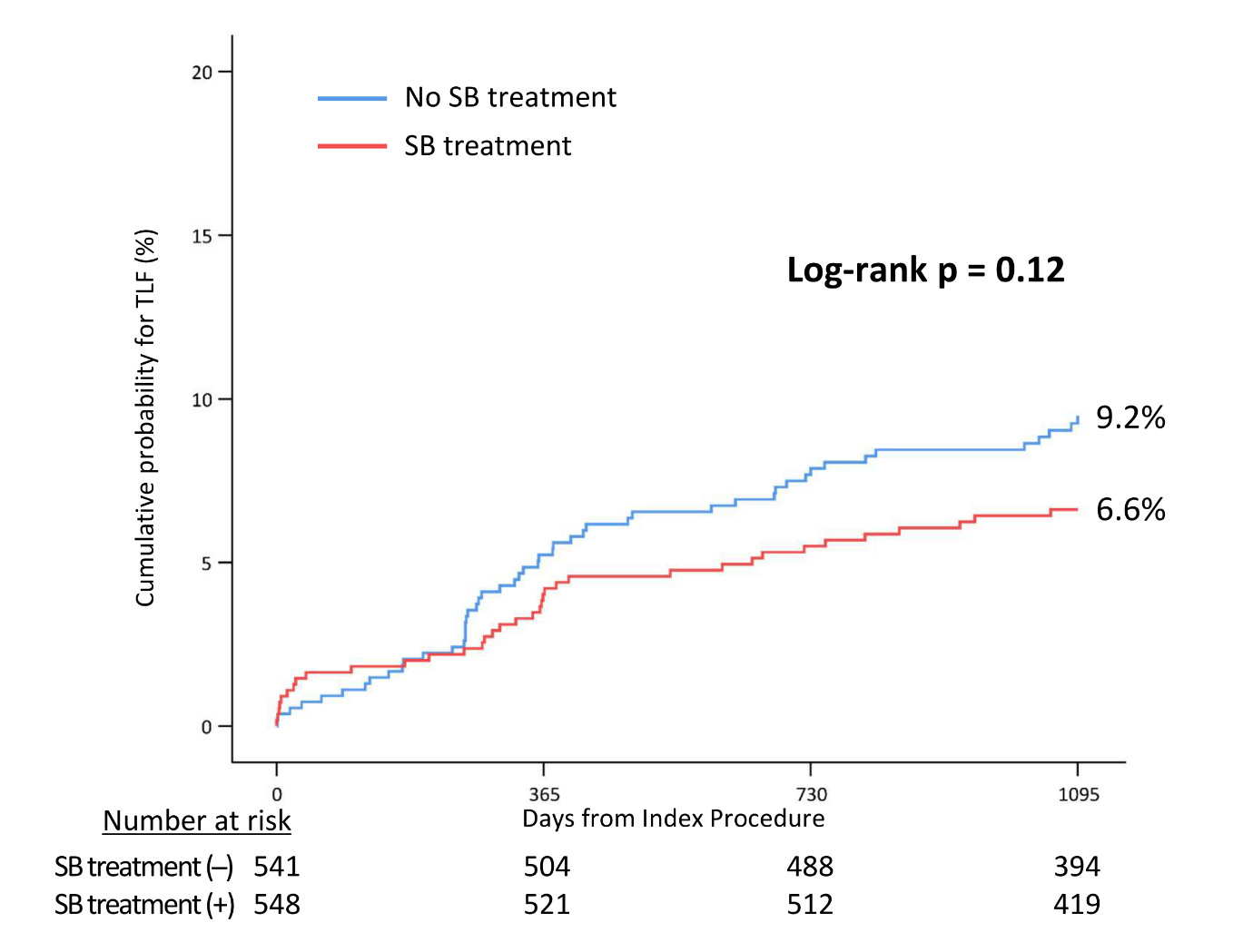
**

TLF, target lesion failure; SB, side-branch.

TLF is a composite of cardiac death, target-vessel myocardial infarction, and target-lesion revascularization.

**Figure S2. Kaplan-Meier curve for 3-year individual outcomes according to side-branch treatment.**

**
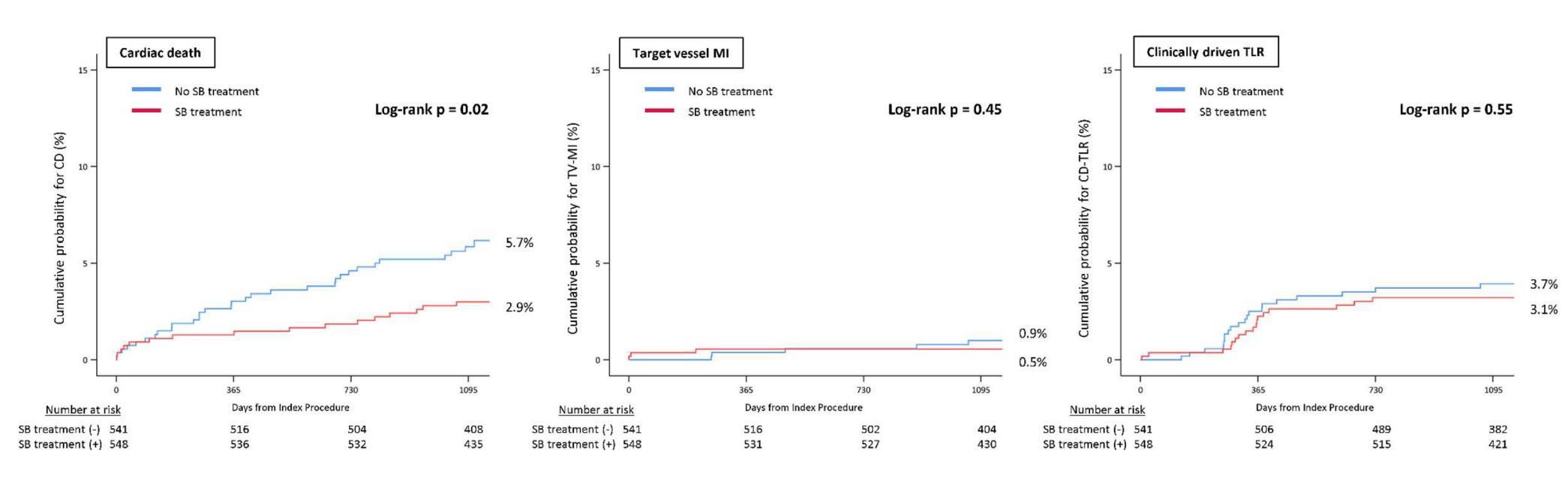
**

MI, myocardial infarction; TLR, target lesion revascularization; SB, side-branch.

**Figure S3. Kaplan-Meier curve for 3-year definite/probable stent thrombosis according to side-branch treatment.**

**

**

SB, side-branch.
